# NT-proBNP and Major Adverse Cardiovascular Events in Patients with ST-Segment Elevation Myocardial Infarction Who Received Primary Percutaneous Coronary Intervention: A Prospective Cohort Study

**DOI:** 10.1155/2021/9943668

**Published:** 2021-11-02

**Authors:** Zuoan Qin, Yaoyao Du, Quan Zhou, Xuelin Lu, Li Luo, Zhixiang Zhang, Ning Guo, Liangqing Ge

**Affiliations:** ^1^Department of Cardiology, The First People's Hospital of Changde City, Changde 415003, China; ^2^Department of the First Clinical College, Jinan University School of Medicine, Guangzhou 510632, China; ^3^Jishou University, Jishou 416000, China; ^4^Department of Science and Education, The First People's Hospital of Changde City, Changde 415003, China; ^5^Department of Pathology, The First People's Hospital of Changde City, Changde 415003, China

## Abstract

**Background:**

The prognostic significance of the amino-terminal fragment of the prohormone brain-type natriuretic peptide (NT-proBNP) in patients with ST-segment elevation myocardial infarction (STEMI) after percutaneous coronary intervention (PCI) has not been fully elucidated. Major adverse cardiovascular events (MACEs) are clinically viable indicators for the accurate, rapid, and safe evaluation of patients with STEMI. This study was designed to investigate the relationship between NT-proBNP levels and the occurrence of short-term MACEs in patients with STEMI who underwent emergency PCI.

**Methods:**

This prospective cohort study included 405 patients with STEMI aged 20–90 years who underwent emergency PCI at the First People's Hospital of Changde City from April 6, 2017, to May 31, 2019. Stent thrombosis, reinfarction, congestive heart failure, unstable angina, and cardiac death were considered as MACEs in this study. The target-independent and -dependent variables were NT-proBNP at baseline and MACE, respectively.

**Results:**

There were 28.25% of MACEs. Age, number of implanted stents, Killip class, infarction-related artery, applied intra-aortic balloon pump (IABP), creatine kinase (CK) peak value, CK-MB peak value, TnI peak value, and ST-segment resolution were independently associated with MACE (*P* < 0.05). In a multivariate model, after adjusting all potential covariates, Log2 NT-proBNP levels remained significantly associated with MACE, with an inflection point of 11.66. The effect sizes and confidence intervals of the left and right sides of the inflection point were 1.07 and 0.84–1.36 (*P*=0.5730) and 3.47 and 2.06–5.85 (*P* < 0.0001), respectively.

**Conclusions:**

In patients with STEMI who underwent PCI, Log2 NT-proBNP was positively correlated with MACE within 1 month when the Log2 NT-proBNP was >11.66 (NT-proBNP >3.236 pg/mL).

## 1. Introduction

The amino-terminal fragment of the prohormone brain-type natriuretic peptide (NT-proBNP) is synthesized by ventricular myocytes. Its release is stimulated by cardiomyocyte stretching. Stretching is caused by pressure or volume overload in the heart. NT-proBNP release is stimulated by myocardial ischemia [1]. NT-proBNP is used as a biomarker to predict both short- and long-term mortality in patients with the acute coronary syndrome (ACS), and it is closely linked to the levels of myocardial ischemia and cardiac function in ACS patients [2]. The term “major adverse cardiovascular events” (MACEs) has no concrete definition, but over time, various definitions have been used in cardiovascular research with MACE selected as the primary or secondary endpoint. It has been defined by various authors since the mid-1990s to include an overlapping range of adverse events [3, 4]. In different studies, MACE includes heart failure (HF), recurrent angina, nonfatal reinfarction, rehospitalization for cardiovascular-related illness, and repeat percutaneous coronary intervention (PCI) [5]. It can also include unscheduled coronary revascularization, stroke, reinfarction, and all-cause death mortality [6]. However, patients with ST-segment elevation myocardial infarction (STEMI) have various risks of developing adverse cardiovascular events [7]. MACE is a clinically viable parameter for the accurate, rapid, and safe evaluation of myocardial perfusion [8]. The prognostic role of NT-proBNP in STEMI has been rarely reported. The relationship between NT-proBNP and MACE has not previously been investigated in patients with STEMI undergoing emergency PCI. The objective of this study was to investigate the association between NT-proBNP levels and MACE and to assess the clinical significance of short-term MACE in patients with STEMI who underwent emergency PCI.

## 2. Materials and Methods

### 2.1. Study Design

We conducted a prospective cohort study to assess the relationship between NT-proBNP and MACEs; the target-independent variable was NT-proBNP, and the dependent variable was any MACE that occurred within 1 month.

### 2.2. Study Population

From April 6, 2017, to May 31, 2019, 405 patients from The First People's Hospital of Changde City met the diagnostic criteria of STEMI based on the 2014 ESC/EACTS guidelines for the diagnosis and treatment of acute STEMI [9]. STEMI is defined as follows: evidence of myocardial damage (defined as elevated cardiac troponin levels), accompanied by persistent chest discomfort or symptoms of ischemia, and ST-segment elevation in at least two adjacent leads of the ECG.

All patients underwent emergency PCI in the first 12 h after admission, with the successful opening of the infarction-related artery (IRA). Inclusion criteria were as follows: (1) they should be according to the Chinese Medical Association “Diagnosis and Treatment of ASTEMI” in the 2014 ESC/EACTS guidelines for the diagnosis and treatment of acute STEMI; (2) onset time was less than 12 h; (3) emergency CAG was performed for acute myocardial infarction; (4) the patients should agree to be followed up. The exclusion criteria were as follows: (1) history of HF before emergency PCI; (2) history of major trauma, surgery, or active gastrointestinal hemorrhage in the past six months; (3) history of severe liver or kidney damage, malignant tumors, and stroke; (4) aortic aneurysm or aortic dissection; (5) significantly high blood pressure and unsatisfactory drug control, hypertensive crisis, and unstable condition; and (6) allergy to contrast agents.

This study was performed with the consent of the Bioethics Committee in accordance with the Declaration of Helsinki and was approved by The First People's Hospital of Changde City, No. 2020-001-01.

### 2.3. Measurement of NT-proBNP

All STEMI patients who met the inclusion criteria completed the NT-proBNP investigation, which was performed using 5 mL of venous blood and electrochemical luminescence immunoassay (ECLIA, NT-proBNP, Roche Diagnostics, Mannheim, Germany) within 2 h after emergency PCI in the laboratory. The measurement range defined by the detection lower limit and the maximum value of the main curve was 5–35,000 pg/mL.

### 2.4. Follow-Up MACE Status

Follow-up was performed by phone or in outpatient clinics. We recorded the MACE status of patients who were discharged from the hospital within 1 month. MACE is defined as a composite of all-cause death, stent thrombosis, reinfarction, congestive HF, and angina pectoris.

### 2.5. Statistical Analysis

Serum NT-proBNP concentration was expressed in pg/mL. We examined the distribution of serum NT-proBNP concentration in patients with STEMI; it was strongly skewed to the right; therefore, it was transformed to the Log 2 scale (Log2 NT-proBNP) for analysis. Continuous variables were presented in two forms. In the first form, continuous variables with normal distribution are expressed as mean ± standard deviation. In the second form, continuous variables with a skewed distribution are presented as medians (Q1–Q3). Categorical variables are expressed as frequencies or percentages. We used *χ*^2^ (categorical variables), one-way ANOVA test (normal distribution), or Kruskal–Wallis H test (skewed distribution) to test for differences among the Log2 NT-proBNP groups (tertiles). The entire data analysis process was divided into two steps. Step 1: univariate and multivariate linear regression analyses were performed. We constructed three models: in model 1, no covariates were adjusted; in model 2, only age and sex were adjusted; in model 3, age, sex, and other covariates presented in Table 1 were adjusted. Step 2: to address the nonlinearity of the relationship between NT-proBNP and MACE, a generalized additive model and smooth curve fitting (penalized spline method) were conducted. If nonlinearity was detected, we first calculated the inflection point using the recursive algorithm and then constructed a two-piecewise linear regression on both sides of the inflection point. Moreover, the best-fit model was determined based on the *P* values for the log-likelihood ratio test. All analyses were performed using the statistical software packages *R* (http://www.R-project.org, *R* Foundation) and EmpowerStats (http://www.empowerstats.com, X&Y Solutions, Inc., Boston, MA). Statistical significance was set at *P* < 0.05.

## 3. Results

### 3.1. Baseline Characteristics of the Selected Participants

**Figure 1:**
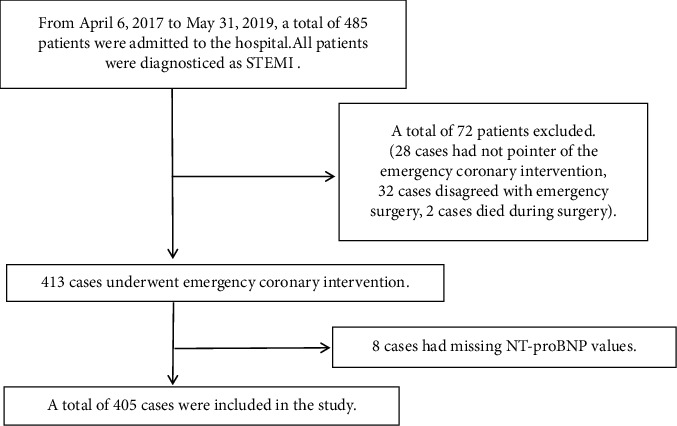
A flowchart on how participants were selected for the final analysis.

A total of 405 participants were selected for the final data analysis (see Figure 1 for a flowchart). The average age of the selected participants was 60.44 ± 12.23 years, and 82.72% of the participants were men. The overall prevalence of MACEs within one month was 28.15% in the study population, including stent thrombosis (*n* = 1), reinfarction (*n* = 11), congestive HF (*n* = 90), angina pectoris (*n* = 13), and cardiac death (*n* = 20). Baseline patient characteristics are summarized in Table 1. Among the groups of patients with different Log2 NT-proBNP levels, there were no statistically significant differences in terms of hyperlipidemia, diabetes, time from admission to balloon dilatation, number of implanted stents, number of used numbers, pre-PCI TIMI grade, post-PCI TIMI grade, IRA, thrombus aspiration catheter use, or use of a temporary pacemaker. Patients with high Log2 NT-proBNP levels (group T3) were significantly older, had a longer time from symptom onset to balloon dilatation, and had higher CK-MB, CK, and TnI peak values than those in groups T1 and T2. The proportion of male participants in group T3 was significantly lower than that in groups T1 and T2. The proportion of participants with TIMI grades 3 and 4 in group T3 was significantly higher than that in groups T1 and T2. The proportion of participants who had an intra-aortic balloon pump (IABP) inserted was significantly higher in group T3 than that in groups T1 and T2. The proportion of participants with ST-segment resolution in group T3 was significantly lower than that in groups T1 and T2.

### 3.2. Univariate Analysis

The results of the univariate analysis are presented in Table 2. These results showed that age, number of implanted stents, Killip class, IRA, insertion of IABP, CK peak value, CK-MB peak value, TnI peak value, NT-proBNP, and ST-segment resolution showed correlation with higher MACE.

We also found that sex, number of balloons used, hypertension, hyperlipidemia, diabetes, smoking, thrombus aspiration catheter use, and use of temporary pacemaker were not associated with Log2 NT-proBNP, whereas the number of implanted stents was negatively associated with higher Log2 NT-proBNP levels. The Killip class was positively correlated with higher Log2 NT-proBNP levels.

### 3.3. Relationship between Log2 NT-proBNP and MACE in Different Models

We used a univariate linear regression model to evaluate the association between Log2 NT-proBNP and MACE. The nonadjusted and adjusted models are presented in Table 3. In the nonadjusted model, the Log2 NT-proBNP levels were positively correlated with MACE (OR, 1.63; 95% confidence interval (CI), 1.40–1.89; *P* < 0.0001). In the adjusted model I (adjusted for age and sex), the result remained significant (OR, 1.60; 95% CI, 1.37–1.86; *P* < 0.0001). In adjusted model II (adjusted for age, sex, number of implanted stents, number of stents used, pre-PCI TIMI grade, hypertension, hyperlipidemia, diabetes, smoking, time from admission to balloon dilatation, Killip class, post-PCI TIMI grade, and TIMI risk score), the results remained significant (OR, 1.50; 95% CI, 1.26–1.78; *P* < 0.0001).

### 3.4. Analysis of Nonlinear Relationship

**Figure 2:**
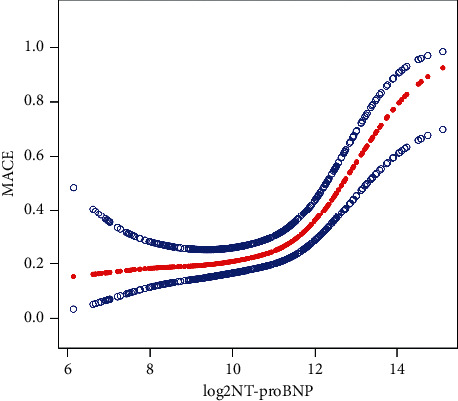
The nonlinear relationship between Log2 NT-proBNP and MACE.

In this study, we found that the relationship between Log2 NT-proBNP and MACE was nonlinear (Figure 2) after adjusting for sex, age, number of implanted stents, number of used balloons, hypertension, hyperlipidemia, diabetes, smoking, time from symptom onset to balloon dilatation, time from admission to balloon dilatation, Killip class, pre-PCI TIMI grade, post-PCI TIMI grade, and TIMI risk score. Using a two-piecewise linear regression model, we calculated the inflection point as 11.66 (NT-proBNP = 3,236 pg/mL; 111 patients had NT-proBNP >3,236 pg/mL). The effect size and CI on the right side of this model were 3.45 and 2.06–5.85 (*P* < 0.0001). However, we did not observe any relationship between NT-proBNP and MACE to the left of this inflection point (1.07) (0.84–1.36, *P*=0.5730) (Table 4).

## 4. Discussion

In the early stage of AMI, ventricular diastolic and systolic function declines sharply, myocardial ischemia at the infarcted site induced rapid release of BNP, and serum NT-proBNP concentration was significantly increased. Therefore, NT-proBNP levels could reflect ventricular load, infarct size, and left ventricular dysfunction.

Emergency PCI was used to unblock the stenosis of the coronary artery lumen by cardiac catheterization technology so that the blood flow in the lesion could be restored in a timely manner, the blood flow could be distributed normally, the occlusion could be relieved, and the myocardial blood perfusion could be further improved. This procedure had a preventive effect on the remodeling of the left ventricle, which would lead to an increase in the content of active substances secreted by the heart and a decrease in the level of plasma B-type brain natriuretic peptide. The 2020 ESC Guidelines for the management of acute coronary syndrome in patients presenting without persistent ST-segment elevation states that the use of BNP or NT-proBNP plasma concentrations should be considered to gain prognostic information [3, 10, 11]. However, our study also found that the NT-proBNP levels have clinical significance in predicting the occurrence of MACEs in patients with STEMI within one month after undergoing emergency PCI. BNP and NT-proBNP are useful prognostic markers in patients with acute decompensated HF, chronic stable HF, ACS, and acute myocardial infarction (AMI) [12, 13]. However, a recent study has shown that the short-term NT-proBNP level is a powerful prognostic marker for all-cause mortality and MACE in patients with AMI [14–16]. In the present study, we used the NT-proBNP value to predict the likelihood of the patient developing MACE within 1 month after discharge. As a result, the present study was conducted to examine the relationship between Log2 NT-proBNP and MACE. As shown in the fully adjusted model, Log2 NT-proBNP was associated with MACE, even after sensitivity analysis. However, we also found a nonlinear relationship between Log2 NT-proBNP and MACE.

We searched for keywords for “NT-proBNP,” “major adverse cardiovascular events,” and “STEMI” in PubMed. There were 23 scientific papers at the end of May 2019, but only six were related to our study. In a recent study of 1,093 Chinese patients with STEMI, Wang et al. [17] found that hs-CRP and NT-proBNP on admission reinforced the predictive power of the Korea Acute Myocardial Infarction Registry (KAMIR) score for long-term adverse cardiovascular events in Chinese patients with STEMI. In another study, emergency PCI resulted in lower plasma NT-proBNP levels and incidence of MACE and better early exercise tolerance than elective PCI or drug treatment; the inference was that lower plasma NT-proBNP levels predicted a better prognosis [18]. Other similar studies have found the same result. For example, in a study by Dai et al. [19], 345 patients with stable coronary artery disease (CAD) were recruited after successful PCI. They found that NT-proBNP and CRP levels were the strongest predictors of MACE. In another study of patients with AMI, Sim et al. [20] found that increased hs-CRP and NT-proBNP levels 24 h after AMI reflected the extent of and reaction to myocardial damage. In a study of BNP and AMI, Lee et al.[14]. found that the combination of the short-term follow-up of BNP level and the initial BNP level could better predict all-cause mortality, and we found that there was a nonlinear relationship and an important turning point (Log2 NT-proBNP = 11.66, NT-proBNP = 3,236 pg/mL) between Log2 NT-proBNP and MACEs in the Chinese population. This can be explained by the fact that elevated levels of early NT-proBNP are associated with acute phase responses from early myocardial necrosis after AMI.

In our study, we found that there was a nonlinear relationship and an important turning point (Log2 NT-proBNP = 11.66, NT-proBNP = 3,236 pg/mL) in the Chinese population between Log2 NT-proBNP and MACEs. When Log2 NT-proBNP was <11.66 (NT-proBNP ≤ 3,236 pg/mL), no statistically significant relationship was found between Log2 NT-proBNP and MACE. However, when the Log2 NT-proBNP was >11.66 (NT-proBNP >3,236 pg/mL), Log2 NT-proBNP was positively correlated with MACE. The greater the Log2 NT-proBNP value, the greater the probability of MACE. Furthermore, in a study by Valente et al. [21], the Kaplan–Meier curves in the PCI studies of 198 patients with STEMI showed that patients who died at follow-up had the highest NT-proBNP quartiles. Their conclusions are consistent with our finding that the larger the NT-proBNP value, the greater the significance of the prediction. This may be because elevated levels of early NT-proBNP are associated with acute phase changes related to early myocardial necrosis after AMI. Most patients had good recovery of coronary blood flow, and emergency PCI improved blood perfusion of injured cardiomyocytes. Within a certain range, NT-proBNP does not cause MACEs. However, studies have shown that approximately 30% of patients with STEMI do not achieve effective myocardial reperfusion after PCI [22]. Once the coronary event has occurred with resulting slow blood flow/no reflow, the damaged myocardium is not well restored, and this would lead to an obvious increase in NT-proBNP levels and increase the probability of MACEs.

The clinical value of this study is as follows. (1) To the best of our knowledge, this is the first observation of an independent association between NT-proBNP and MACE in Chinese patients with STEMI who have undergone emergency PCI. (2) The results of this study should be useful in future studies to determine whether the NT-proBNP level in patients with STEMI has predictive power for MACE occurrence after emergency PCI.

Our study had several strengths. (1) We solved the rationale behind the nonlinearity of NT-proBNP and MACE in the present study. (2) This is an observational study and would be likely to lead to misinterpretation. We used strict statistical adjustments to minimize residual confounders. (3) Using the two-stage linear regression model, we calculated the inflection point as 11.66. To the right of the inflection point, the effect size, 95% CI, and *P* value were 3.47, 0.86–1.37, and <0.0001, respectively.

There are some limitations to this study, including the following. (1) In this study, our research participants were Chinese patients with STEMI. Therefore, there is a deficiency in the universality and extrapolation of the results. (2) Because we excluded patients with acute non-ST-segment elevation myocardial infarction, unstable angina, or some patients with STEMI who had HF before emergency PCI, the study was not applicable to these patients.

## 5. Conclusions

Log2 NT-proBNP was positively correlated with MACE within 1 month when Log2 NT-proBNP was >11.66 (NT-proBNP > 3,236 pg/mL) in hospitalized patients who had emergency coronary intervention (PCI) for STEMI.

## Figures and Tables

**Table 1:** Baseline characteristics of the participants.

NT-proBNP (pg/mL, min to max)	Low (T1) (70–891.3)	Middle (T2) (896–2,538)	High (T3) (2,540–35,000)
Number	135	135	135
Age (years, mean ± sd)	57.27 ± 11.73	59.16 ± 11.33	64.88 ± 12.39

*Sex (n (%))*			
Female	15 (11.11%)	22 (16.3%)	33 (24.44%)
Male	120 (88.89%)	113 (83.70%)	102 (75.56%)

*Hypertension (n (%))*			
No	62 (45.93%)	68 (50.37%)	47 (34.81%)
Yes	73 (54.07%)	67 (49.63%)	88 (65.19%)

*Hyperlipidemia (n (%))*			
No	90 (66.67%)	88 (65.19%)	92 (68.15%)
Yes	45 (33.33%)	47 (34.81%)	43 (31.85%)

*Diabetes (n (%))*			
No	113 (83.7%)	105 (77.78%)	107 (79.26%)
Yes	22 (16.3%)	30 (22.22%)	28 (20.74%)

*Prior MI (n (%))*			
No	131 (97.04%)	130 (96.3%)	126 (93.33%)
Yes	4 (2.96%)	5 (3.7%)	9 (6.67%)

*Smoking (n (%))*			
No	67 (49.63%)	85 (62.96%)	86 (63.7%)
Yes	68 (50.37%)	50 (37.04%)	49 (36.3%)

*Killip class (n (%))*			
I	125 (92.59%)	115 (85.19%)	89 (65.93%)
II	8 (5.93%)	11 (8.15%)	23 (17.04%)
III	1 (0.74%)	6 (4.44%)	15 (11.11%)
IV	1 (0.74%)	3 (2.22%)	8 (5.93%)
Time from symptom onset to balloon dilatation (h; median, Q1–Q3)	6 (4–8)	6 (4.5–10)	7 (5–11)
Time from admission to balloon dilatation (min; median, Q1–Q3)	73 (52.5–96)	72 (51–89)	73 (54–98)

*Number of implanted stents (n (%))*			
PTCA	7 (5.19%)	3 (2.22%)	6 (4.44%)
1-stent	102 (75.56%)	101 (74.81%)	88 (65.19%)
2-stent	24 (17.78%)	28 (20.74%)	35 (25.93%)
3-stent	2 (1.48%)	3 (2.22%)	6 (4.44%)

*Number of used balloons (n (%))*			
1-balloon	111 (82.22%)	114 (84.44%)	104 (77.04%)
2-balloon	23 (17.04%)	18 (13.33%)	31 (22.96%)
3-balloon	1 (0.74%)	3 (2.22%)	0 (0%)

*Pre-PCI TIMI grade (n (%))*			
TIMI0	95 (70.37%)	95 (70.37%)	112 (82.96%)
TIMI1	20 (14.81%)	23 (17.04%)	14 (10.37%)
TIMI2	20 (14.81%)	17 (12.59%)	9 (6.67%)

*Post-PCI TIMI grade (n (%))*			
TIMI0	2 (1.49%)	1 (0.74%)	0 (0%)
TIMI1	0 (0%)	0 (0%)	1 (0.74%)
TIMI2	4 (2.99%)	8 (5.93%)	9 (6.67%)
TIMI3	128 (95.52%)	126 (93.33%)	125 (92.59%)

*Infarction-related artery (n (%))*			
LM	2 (1.48%)	3 (2.22%)	4 (2.96%)
LAD	55 (40.74%)	66 (48.89%)	74 (54.81%)
LCX	19 (14.07%)	14 (10.37%)	12 (8.89%)
RCA	59 (43.7%)	52 (38.52%)	45 (33.33%)

*Use of thrombus aspiration catheter (n (%))*			
No	128 (94.81%)	122 (90.37%)	128 (94.81%)
Yes	7 (5.19%)	13 (9.63%)	7 (5.19%)

*Applied IABP (n (%))*			
No	132 (97.78%)	128 (94.81%)	118 (87.41%)
Yes	3 (2.22%)	7 (5.19%)	17 (12.59%)

*Temporary pacemaker (n (%))*			
No	119 (88.15%)	113 (83.70%)	115 (85.19%)
Yes	16 (11.85%)	22 (16.30%)	20 (14.81%)

*ST-segment resolution (n (%))*			
No	22 (16.3%)	34 (25.19%)	52 (38.52%)
Yes	113 (83.7%)	101 (74.81%)	83 (61.48%)
CK peak value (U/L; median, Q1–Q3)	1,874 (943.5–3,314.65)	2,544 (1,491.5–4,349.95)	3,032 (1,719–5,258.00)
CK-MB peak value (U/L; median, Q1–Q3)	166.6 (78.1–291)	227.6 (117.35–356.9)	240.9 (155.7–439.4)
TnI peak value (U/L, median, Q1–Q3)	5 (1.3–14.4)	9.52 (2.4–22.67)	12.00 (4.2–25)
NT-proBNP (pg/mL; median, Q1–Q3)	485.5 (285–674.4)	1,560 (1,139–1,962.5)	4,896 (3,510–7,805)

*Note.P* ^*∗*^ was calculated using the Kruskal–Wallis H test. We divided the NT-proBNP values of all patients into three groups using the tertiles method: low, medium, and high, namely, T1, T2, and T3. LM, left main; LAD, left anterior descending; LCX, left circumflex artery; RCA, right coronary artery; IABP, intra-aortic balloon pump; PTCA, percutaneous transluminal coronary angioplasty; CK, creatine kinase; CK-MB, creatine kinase isoenzyme-MB.

**Table 2:** MACE of univariate analysis.

	Statistics	YY
*Sex (n (%))*		
Female	70 (17.28%)	1.0
Male	335 (82.72%)	0.98 (0.55, 1.73) 0.9310
Age (year)	60.44 ± 12.23	1.03 (1.01, 1.05) 0.0021

*Number of implanted stents (n (%))*		
PTCA	16 (3.95%)	1.0
1-stent	291 (71.85%)	0.22 (0.08, 0.62) 0.0045
2-stent	87 (21.48%)	0.23 (0.07, 0.70) 0.0095
3-stent	11 (2.72%)	0.13 (0.02, 0.84) 0.0315

*Number of used balloons (n (%))*		
1-balloon	329 (81.23%)	1.0
2-balloon	72 (17.78%)	0.90 (0.50, 1.59) 0.7093
3-balloon	4 (0.99%)	0.83 (0.09, 8.11) 0.8752

*Hypertension (n (%))*		
No	177 (43.70%)	1.0
Yes	228 (56.30%)	1.34 (0.86, 2.08) 0.1953

*Hyperlipidemia (n (%))*		
No	270 (66.67%)	1.0
Yes	135 (33.33%)	0.63 (0.39, 1.02) 0.0619

*Diabetes (n (%))*		
No	325 (80.25%)	1.0
Yes	80 (19.75%)	1.12 (0.65, 1.91) 0.6811

*Smoking (n (%))*		
No	238 (58.77%)	1.0
Yes	167 (41.23%)	0.66 (0.42, 1.04) 0.0732
Time from symptom onset to balloon dilatation (h)	6.94 ± 3.01	1.05 (0.98, 1.13) 0.1650
Time from admission to balloon dilatation (min)	75.69 ± 30.32	1.00 (0.99, 1.01) 0.5895

*Killip class (n (%))*		
I	329 (81.23%)	1.0
II	42 (10.37%)	2.47 (1.26, 4.83) 0.0081
III	22 (5.43%)	9.69 (3.66, 25.67) <0.0001
IV	12 (2.96%)	18.17 (3.89, 84.82) 0.0002

*Pre-PCI TIMI grade (n (%))*		
TIMI0	302 (74.57%)	1.0
TIMI1	57 (14.07%)	0.99 (0.53, 1.83) 0.9629
TIMI2	46 (11.36%)	0.35 (0.14, 0.85) 0.0204

*Post-PCI TIMI grade (n (%))*		
TIMI0	3 (0.74%)	1.0
TIMI1	1 (0.25%)	-
TIMI2	21 (5.20%)	1.50 (0.12, 19.24) 0.7554
TIMI3	379 (93.81%)	0.75 (0.07, 8.32) 0.8120

*Infarction-related artery (n (%))*		
LAD	195 (48.15%)	1.0
LM	9 (2.22%)	8.07 (1.63, 40.00) 0.0106
LCX	45 (11.11%)	0.75 (0.35, 1.57) 0.4405
RCA	156 (38.52%)	0.72 (0.44, 1.16) 0.1729

*Use of thrombus aspiration catheter (n (%))*		
No	378 (93.33%)	1.0
Yes	27 (6.67%)	1.55 (0.69, 3.49) 0.2909

*Applied IABP (n (%))*		
No	378 (93.33%)	1.0
Yes	27 (6.67%)	5.87 (2.55, 13.51) <0.0001

*Temporary pacemaker (n (%))*		
No	347 (85.68%)	1.0
Yes	58 (14.32%)	0.97 (0.52, 1.80) 0.9181
CK peak value (U/L)	3,089.71 ± 2,484.25	1.00 (1.00, 1.00) 0.0021
CK-MB peak value (U/L)	270.88 ± 246.23	1.00 (1.00, 1.00) 0.0051
TnI peak value (U/L)	11.22 ± 9.55	1.03 (1.01, 1.06) 0.0040

*ST-segment resolution (n (%))*		
No	108 (26.67%)	1.0
Yes	297 (73.33%)	0.50 (0.31, 0.80) 0.0041
NT-proBNP (pg/mL)	3,050.54 ± 4,565.76	1.00 (1.00, 1.00) <0.0001

*Note*. -: the model failed due to the small sample. ST-segment resolution: ST elevation of the IRA after surgery from that before surgery, which was then divided by the ST elevation before surgery.

**Table 3:** Relationship between Log NT-ProBNP and MACE in different models.

Variable	Nonadjusted^1^ (*β*, 95% CI, *P* value)	Adjusted I (*β*, 95% CI, *P* value)	Adjusted II (*β*, 95% CI, *P* value)
Log NT-proBNP (pg/mL)	1.63 (1.40, 1.89, <0.0001)	1.60 (1.37, 1.86, <0.0001)	1.50 (1.26, 1.78, <0.0001)

Log NT-proBNP (pg/mL) (quartile)			
T1	1.0	1.0	1.0
T2	1.55 (0.84, 2.88, 0.1636)	1.53 (0.82, 2.85, 0.1793)	1.24 (0.63, 2.46, 0.5390)
T3	4.75 (2.67, 8.44, <0.0001)	4.38 (2.42, 7.93, <0.0001)	3.31 (1.69, 6.48, 0.0005)
P For trend	<0.0001	<0.0001	<0.0001

^1^Nonadjusted model. Adjusted model I: adjusted for age and sex. Adjusted model II: adjusted for age, sex, number of implanted stents, number of the used balloons, pre-PCI TIMI grade, hypertension, hyperlipidemia, diabetes, smoking, time from admission to balloon dilatation, Killip class, post-PCI TIMI grade, and TIMI risk score.

**Table 4:** Piecewise linear regression model was used to detect the association of Log2 NT-ProBNP and MACE according to the Log NT-proBNP cutoff points.

Cutoff points	Hazard ratio	95% CI	*P* value
≤11.66	1.07	0.84–1.36	0.5730
>11.66	3.47	2.06–5.85	<0.0001

Adjusted variables: sex, age, number of implanted stents, number of balloons used, hypertension, hyperlipidemia, diabetes, smoking, time from symptom onset to balloon dilatation, time from admission to balloon dilatation, Killip class, post-PCI TIMI grade, and TIMI risk score.

## Data Availability

The data used to support the findings of this study are available from the corresponding author upon request.

## Abbreviations

NT-proBNP: Amino-terminal fragment of the prohormone brain-type natriuretic peptide
STEMI: ST-segment elevation myocardial infarction
PCI: Percutaneous coronary intervention
IABP: Intra-aortic balloon pump
ACS: Acute coronary syndrome
CAD: Coronary artery disease
LM: Left main
LAD: Left anterior descending
LCX: Left circumflex artery
RCA: Right coronary artery
PTCA: Percutaneous transluminal coronary angioplasty
CK: Creatine kinase
CK-MB: Creatine kinase isoenzyme-MB.
